# Maximum recruitment strategy revealed efficiency and a larger recruitable lung in a prospective series of early ARDS patients

**DOI:** 10.1186/cc10187

**Published:** 2011-06-22

**Authors:** GFJ Matos, F Stanzani, RH Passos, MF Fontana, R Albaladejo, RE Caserta, DCB Santos, JB Borges, MBP Amato, CSV Barbas

**Affiliations:** 1 Hospital Israelita Albert Einstein, São Paulo - SP, Brazil

## Introduction

A recent meta-analysis demonstrated that higher levels of PEEP were associated with improved survival among the subgroup of patients with ARDS. The maximum recruitment strategy (MRS) guided by thoracic CT scan is capable of reversing alveolar collapse almost completely, allowing PEEP titration to sustain lungs almost fully open, homogenizing tidal ventilation and possibly reducing ventilator-induced lung injury.

## Objective

To test the efficiency, feasibility and side effects of MRS; to compare the amount of non-aerated tissue during MRS and calculate lung recruitability.

## Methods

A case series report in a general medical/surgical private and academic ICU with 42 beds at Albert Einstein Hospital, São Paulo, Brazil. Fifty-one severe ARDS patients were included. Early and severe ARDS patients were submitted to MRS guided by thoracic CT. The protocol consisted of two parts: recruitment phase to calculate the opening pressure (PEEP 10 to 45 cmH_2_O and constant driving pressure 15 cmH_2_O); PEEP titration phase (PEEP 25 to 10 cmH_2_O) to maintain the lungs open. Patients were followed until hospital discharge or death.

## Results

Fifty-one severe ARDS patients were included and followed, of whom 84% had primary ARDS. The median maximum recruitment PEEP level was 45 (IQR: 43 to 45) cmH_2_O and the median maximum recruitment plateau pressure was 60 (IQR: 58 to 60) cmH_2_O, and the median titrated PEEP after MRS was 25 (IQR: 25 to 25) cmH_2_O. Median global nonaerated parenchyma decreased significantly from 53.6% (IQR: 42.5 to 62.4) to 12.7% (IQR: 4.9 to 24.2) (p_2_/FiO_2 _ratio increased from 125 (IQR: 86 to 164) to 307 (IQR: 236 to 373)) (*P *< 0.01).

## Conclusion

The MRS was an efficient, feasible and safe ventilatory strategy to reverse nonaerated lung and hypoxemia in early and severe ARDS patients with multiple organ failure, revealing a larger recruitable lung. No major complications except for transitory changes in blood pressure were noted.

